# Mental Health Impacts of Wildfire, Flooding and COVID-19: on educators: A Comparative Study

**DOI:** 10.1192/j.eurpsy.2023.2013

**Published:** 2023-07-19

**Authors:** B. Agyapong, E. Eboreime, R. Shalaby, H. Pazderka, G. Obuobi-Donkor, M. Adu, W. Mao, F. Oluwasina, E. Owusu, A. Greenshaw, V. Agyapong

**Affiliations:** 1 Department of Psychiatry, University of Alberta, Edmonton, Canada

## Abstract

**Introduction:**

Fort McMurray, a city in northern Alberta, Canada, has experienced multiple traumas in the last five years, including the 2016 wildfire, the 2020 floods, and the COVID-19 pandemic. Eighteen months after the wildfire, major depressive disorder (MDD), generalized anxiety disorder (GAD), and Post Traumatic Stress Disorder (PTSD) symptoms were elevated among school board employees in the city.

**Objectives:**

This study aimed to compare employees of the school board and other employees of Fort McMurray in respect to the impact the 2016 wildfires, the 2019 COVID pandemic, and the 2020 floods had on their mental health.

**Methods:**

A quantitative cross-sectional survey was conducted in Fort McMurray from 24 April to 2 June 2021. Online questionnaires were administered through REDCap and were designed to capture socio-demographic characteristics, clinical as well as wildfire, COVID-19, and flooding-related variables. Mental health outcome variables were captured using self-reported standardized assessment scales. Data were analysed with descriptive statistics, Chi-square/Fisher’s Exact tests, and binary regression analysis.

**Results:**

Of the 249 residents who accessed the online survey, 186 completed the survey, giving a response rate of 74.7%. Of these respondents, 93.5% (174) indicated their employment status and were included in the Chi-square analysis. Most of the respondents were female (86.2%, (150)), above 40 years (53.4%, (93)), and were in a relationship (71.3%, (124)). The prevalence values for MDD, GAD and PTSD among respondents were 42.4%, 41.0, and 36.8%, respectively. There was a statistically significant difference between employees of the school board and other employees with respect to likely PTSD prevalence (28% vs. 45%, respectively, *p* < 0.05), although with other factors controlled for, in a binary logistic regression model, employer type did not significantly predict likely PTSD.

**Conclusions:**

The study has established that likely PTSD symptoms were significantly higher in other employees compared to those of school board employees. Greater exposure to the traumatic events and a greater perceived lack of support from other employers might have contributed to the significantly higher prevalence of PTSD in other employees

**Disclosure of Interest:**

None Declared

